# Antimicrobial resistance burden, and mechanisms of its emergence in gut microbiomes of Indian population

**DOI:** 10.3389/frmbi.2024.1432646

**Published:** 2024-07-18

**Authors:** Nisha Chandel, Jeremiah Paul Gorremuchu, Vivek Thakur

**Affiliations:** ^1^ Department of Systems and Computational Biology, University of Hyderabad, Hyderabad, India; ^2^ Department of Biotechnology, Chaitanya Bharathi Institute of Technology, Hyderabad, India

**Keywords:** human gut metagenome, healthy Indian cohorts, antibiotic resistance, mobile genetic elements, horizontal gene transfer

## Abstract

**Introduction:**

The human gut microbiome harbors millions of bacterial species, including opportunistic pathogens, and this microbial community is exposed to antimicrobial agents present in food, the external environment, or drugs. Thus, it increases the risk of commensals being enriched with resistant genes, which may get even transmitted to opportunistic pathogens often with the help of mobile genetic elements. There is limited information about the current burden of resistant genes in the healthy gut microbiome of the Indian population, the latter is not only the largest in the world but is also periodically monitored for the prevalence of antibiotic resistance in clinical samples.

**Methods:**

We analyzed publicly available fecal whole-metagenome shotgun sequencing data from 141 samples from three healthy Indian cohorts for antimicrobial-resistance burden, and their likely transmission modes.

**Results:**

The overall resistance profile showed a higher number of resistance genes against tetracycline, glycopeptide, and aminoglycoside. Out of a total of 188 antimicrobial resistance genes identified in all cohorts, moderately to highly prevalent ones could potentially target seven of the ‘reserve’ group antibiotics (colistin, fosfomycin, Polymyxin). We also observed that geographical location affected the prevalence/abundance of some of the resistance genes. The higher abundance of several tetracycline and vancomycin resistance genes in tribal cohorts compared to the other two urban locations was intriguing. Species *E. coli* had the highest number of resistant genes, and given its relatively modest abundance in gut microbiomes can pose a risk of becoming a hub for the horizontal transfer of resistance genes to others. Lastly, a subset of the resistance genes showed association with several types of mobile genetic elements, which potentially could facilitate their transmission within the gut community.

**Discussion:**

This is a first systematic report on AMR genes in healthy gut microbiome samples from multiple locations of India. While trends for several of the prevalent AMR genes showed similarity with global data, but a few population specific trends need further attention by policy-makers. The association of AMR genes with mobile elements may pose a risk for transmission to other gut bacteria.

## 1 Introduction

Antimicrobial resistance (AMR) occurs when bacteria, fungi, and, viruses do not respond to the antimicrobial drugs, which are designed to either kill them or inhibit their growth. As a result, the infection becomes difficult or sometimes impossible to cure, which leads to severe illness, disease spread, and death (Murray et al., 2022). It is one of the leading health problems of the 21st century, which has claimed around 4.95 million lives in 2019 as estimated by predictive statistical models (Murray et al., 2022) and it could kill 10 million people every year by 2050 (O’Neill, 2016). The resistance mechanism could arise due to mutation in the chromosomal genes or by AMR gene acquisition from the same or different species through a process called horizontal gene transfer (HGT). HGT can occur in any environment where bacterial load is high such as hospital settings, soil, treatment plants, livestock, and human gut microbiome (McInnes et al., 2020).

To use antibiotics safely and effectively, the WHO expert committee developed the AWaRe classification system in 2017. It has three groups: Access, Watch, and Reserve. The ‘Access’ antibiotics are narrow-spectrum in action, are first or second-choice treatments for common infections, and generally have low resistance potential. The ‘Watch’ antibiotics are broader-spectrum antibiotics, generally have higher costs, and are recommended only as first-choice options for more severe infections or for infections where the causative pathogens are more likely to be resistant to Access antibiotics. Reserve antibiotics are the last-choice antibiotics used to treat multidrug-resistant infections (World Health Organization, 2024). Notably, incorporating WHO’s AWaRe classification system in structured antibiotic prescription is crucial for the safety of patients and combating its misuse (Elshenawy et al., 2023).

The diverse human gut microbial community acts as a reservoir of AMR genes (Ghosh et al., 2013; Hu et al., 2013; Bag et al., 2019; Monaghan et al., 2020). Like pathogenic bacteria, gut commensals also have antibiotic resistance potential (Kumar et al., 2017; Bag et al., 2019). Studies on AMR gene prevalence and transmission have often been done on clinical samples and latent sources have not been explored much. The latter can also play an important role in AMR transmission. A systematic gut resistome profiling of 1.4k healthy subjects from twelve countries’ was done by (Qiu et al., 2020). suggests the geographical origin of subjects to be the substantial factor for gut AMR gene composition (Qiu et al., 2020). A higher abundance of AMR genes in healthy gut microbiome of individuals from different nationalities has been reported in the last decade (Ghosh et al., 2013; Hu et al., 2013). Another systematic resistome profiling by analyzing more than 10,000 metagenome samples showed a higher abundance and diversity of latent AMR gens compared to established ones in humans, animals, and their associated environments (Inda-Díaz et al., 2023).

India, despite being a region under watch for AMR, not a single systematic study of AMR prevalence was done on the gut microbiome of healthy subjects, except for (Monaghan et al., 2020), which examined 105 samples from (diarrheal and non-diarrheal from urban and rural locations) central India, but the non-diarrheal samples were again sourced from the hospital (Monaghan et al., 2020). AMR surveillance is being routinely done at the national and global level but it is mostly limited to common pathogens in clinical samples (AMR Surveillance Network, 2021; Murray et al., 2022; Kaur et al., 2024). The resistome profiling of five gut commensals, eight gram-negative enteric pathogens, and investigation of the prevalence of 35 AMR genes in Indian tribes are among the different studies done in India (Kumar et al., 2017; Bag et al., 2019; Sethi et al., 2022).

While the gut microbiota frequently gets exposed to antimicrobials, none of the present studies has systematically looked at the AMR gene load in a healthy Indian population, so the present study aims to profile AMR genes in such group. To achieve this, we utilized and analyzed publicly available Whole Metagenome Shotgun (WGS) data of three cohorts consisting of 141 gut microbiome samples from healthy Indian individuals. The metagenome data was analyzed to assess the prevalence and abundance of AMR genes targeting antibiotics in the WHO AWaRe classification (World Health Organization, 2024). We further examined the common/unique trends of the mode of transmission through HGT, and mechanisms of resistance. Finally, any effect of age, diet, and location or lifestyle on the AMR gene prevalence in healthy gut microbiome was also investigated.

## 2 Methods

### 2.1 Sequence data retrieval of Indian metagenome samples

**Figure 1:**
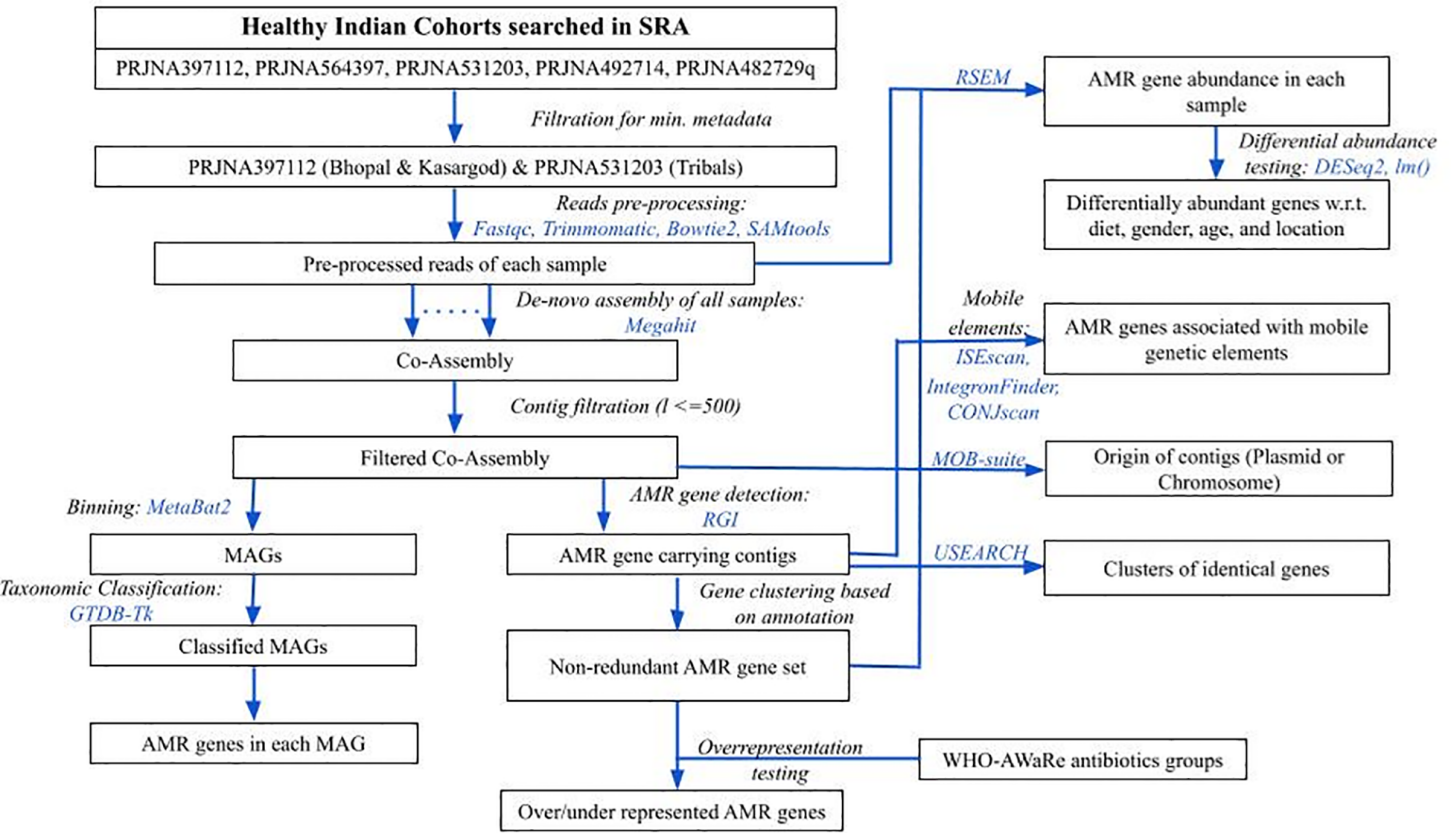
An overview of methodology followed in the analysis. Blue colored text represents the tools used in respective steps.

To obtain gut metagenome samples of Indians, the NCBI Short Read Archive (SRA) database was searched for the keywords “human gut metagenome” with or without additional ones like “India”, and “healthy” (database accessed on March 2023). Results were manually curated for the studies involving WGS sequencing data of individuals without any disease at the sampling time. Samples under Bioproject IDs PRJNA397112 (N=110) (Dhakan et al., 2019), PRJNA531203 (N=31) (Kaur et al., 2020), PRJNA482729 (N=12) (NCBI-Bioproject database), PRJNA492714 (N=12) (Bhushan et al., 2021), and PRJNA564397 (N=47) (Monaghan et al., 2020) were from healthy subjects, so they were further checked for the minimum metadata information namely, age, gender, diet, and location. Since the samples in PRJNA482729 and PRJNA492714 had missing age information, and those in PRJNA482729 also had missing dietary information, so both of them were dropped from the analysis. For the samples in PRJNA564397, although complete metadata information was available, however, the control group was sourced from hospital samples without diarrheal conditions, hence they were also excluded from the study (**Figure 1**).

Finally, a total of 141 healthy gut metagenome samples with consistent metadata information from the remaining two bioproject IDs were considered representative of the Indian population, and their WGS sequencing data were downloaded. Briefly, Bioproject ID PRJNA397112 included 110 samples from two urban locations, namely Bhopal (central India) and Kasargod (south-west coastal city), whereas, PRJNA531203 had 31 samples from three tribal populations (Ladakh, Jaisalmer, and Khargone), which were grouped as one based on their lower beta-diversity values (Singh et al., 2019; Kaur et al., 2020). The average age of all the samples was 30.6 ± 16.29 years. There were 60 females and 81 males who broadly followed an omnivorous (n=100) and vegetarian (n=41) diet (refer to (Chandel et al., 2023) for details).

### 2.2 Quality-control of metagenome data

The downloaded sequence data consisted of only Illumina paired-end reads, which were checked for overall quality using FastQC (version: 0.12.1) (Andrews, 2010). The adapter content and low-quality reads (phred score < 20) were removed using Trimmomatic (version: 0.39) (Bolger et al., 2014). Illumina adaptor sequences provided in the Trimmomatic package were removed. Other key parameters were: Reads were scanned with a 4-base wide sliding window and were cut when the average quality per base dropped below 20. The average number of pre-processed reads and sequence data were ~10 million and ~3 Gbases, respectively (**Supplementary Table S1**). Reads were further filtered for host contamination by first aligning the reads to the human reference genome (genome assembly GRCh38.p14 downloaded from NCBI) using bowtie2 (version: v2.5.3) (Langmead and Salzberg, 2012), followed by extraction of unmapped reads using samtools (version: 1.20) (Li et al., 2009) by specifying SAM-flags for unmapped reads (**Figure 1**).

### 2.3 Co-assembly of the metagenome data, antibiotic resistome detection, and abundance estimation

The pre-processed reads from all samples were pooled together, and a co-assembly was generated using megahit (version: 1.2.9) (Li et al., 2016). Contigs with length less than 500 bp were removed. The antibiotic resistance genes in the entire filtered co-assembly were predicted using the Resistance Gene Identifier (RGI) (version: 6.0.3) with default parameters (Alcock et al., 2023), which uses Prodigal as a gene prediction tool (Hyatt et al., 2010) and Comprehensive Antibiotic Resistance Database (CARD) (version: 4.0.2) as a reference (Alcock et al., 2020). Since the AMR genes predicted by RGI were also functionally annotated, the non-redundant set of AMR genes were obtained based on their functional annotation. The abundance of non-redundant set of observed AMR genes in each sample was estimated by obtaining the count of reads mapped to them using the RSEM tool (version: 1.3.3) in transcripts per million (TPM) (**Figure 1**) (Li and Dewey, 2011). An AMR gene with an abundance of more than 0 was considered present in the sample.

### 2.4 Binning and taxonomic profiling

The filtered co-assembly was binned using metabat2 (version: 2.15) (Kang et al., 2019), and each bin’s quality was determined using checkM (version: 1.2.2) (Parks et al., 2015). Bins henceforth will be called Metagenome Assembled Genomes (MAGs) (**Figure 1**). To retain the majority of the bins, those with completeness >= 50% and contamination <=10% were kept for ongoing analysis as acceptable quality, consistent with the medium genome quality presented by (Bowers et al., 2017). The entire co-assembly was divided into acceptable-quality MAGs, poor-quality MAGs, and co-assembly remained unassigned to any of the MAGs, henceforth, labeled as “unbinned”, which also carried extrachromosomal DNA. The occurrence of AMR-gene-carrying contigs (identified above in section 2.3) was calculated for binned and unbinned contigs sets, and the difference in the proportion between two sets was tested using the 2-sample test for equality of proportions, *prop.test()* in R package (version 4.2.2; r-project.org).

The taxonomy of acceptable quality MAGs was assigned using the GTDB-Tk tool (version: 2.4.0) (Chaumeil et al., 2022), which uses Genome Database Taxonomy (GTDB) information (**Figure 1**). The presence of AMR genes detected in the previous step was confirmed in these groups.

### 2.5 Statistical testing of differentially abundant AMR genes

Differential AMR gene abundance testing for multiple conditions along with the confounders (locations, dietary habits, age, and gender) was done using negative binomial Generalized Linear Model (GLM), as implemented in DESeq2 package (version: 1.42.1) (Love et al., 2014), and also using the log-transformed multiple linear regression model (implemented in *lm*() function of R package), while controlling the effect of covariates (**Figure 1**). The significantly abundant AMR genes common in both approaches were further considered. All other statistical tests were performed in R (version 4.2.2; r-project.org). All the figures were generated using ggplot2 (version: 3.5.0) (Wilkinson, 2011) and VennDiagram (version: 1.7.3) (Chen and Boutros, 2011) packages in R (version 4.2.2; www.r-project.org).

### 2.6 Antibiotics of WHO-AWaRe groups that are targeted by AMR genes

Since AMR genes can target the antibiotics grouped under the WHO-AWaRe classification, so it was tested if any of the WHO-AWaRe groups are overrepresented among the (AMR targeted) antibiotics (**Figure 1**). A list of antibiotics belonging to the WHO-AWaRe group was downloaded from the WHO (World Health Organization, 2024), and the antibiotics targeted only by highly prevalent AMR genes (percentage of samples in which AMR gene is present: >=80), were mapped in the AWaRe list. A contingency table was prepared having the number of antibiotics from the WHO-AWaRe list, belonging (or not belonging) to a particular group, which can (or cannot) be targeted by the AMR genes. Observing the skewed size of the count data (<=5), Fisher’s exact test was used to test the difference in frequency of the antibiotic groups between two lists (*fisher.test*() in R). The parameter “alternative” was set to “less” when the odds ratio of the AWaRe group was less than 1, and “greater” otherwise.

In a related mapping analysis, the antibiotics targeted by moderately or highly prevalent AMR genes (>20%) were mapped only to the ‘Reserve’ group of the AWaRe list, as transmission of AMR genes targeting this group of antibiotics has been of higher concern than others. The frequency distribution of prevalence of such AMR genes was generated using the histogram option with a bin size of 10. Since AMR genes may act against more than one antibiotic, those exclusively acting against the ‘Reserve’ group were distinguished from the remaining ones.

### 2.7 Detection of mobile genetic elements associated with AMR genes

The contigs having any of the AMR genes were also examined to determine whether they are part of any of the mobile genetic elements, potentially facilitating the transmission of AMR genes. Among the mobile elements that were examined included Insertion Sequence (IS) elements/DNA transposons by using ISEscan (version: 1.7.2.3) (Xie and Tang, 2017), integrons by using IntegronFinder (version: 2) (Néron et al., 2022), and Integrative and Conjugative elements (ICEs) or plasmid conjugative elements by using CONJscan (version: 2.0.1) (**Figure 1**) (Cury et al., 2020). Even a partial association (overlap of >=1 nucleotide base) of the AMR gene with the mobile elements was considered for further analysis.

The recent occurrence of the HGT event was examined by identifying identical AMR gene sequences in two or more species using the clustering tool of the USEARCH package (version: 11.0.667) with 100% identity threshold (Edgar, 2010). The contigs of plasmid origin were identified using MOB-suite (version: 3.1.9) (**Figure 1**) (Robertson and Nash, 2018).

## 3 Results

### 3.1 188 AMR genes were identified in the co-assembly of 141 Indian gut metagenome samples

**Figure 2:**
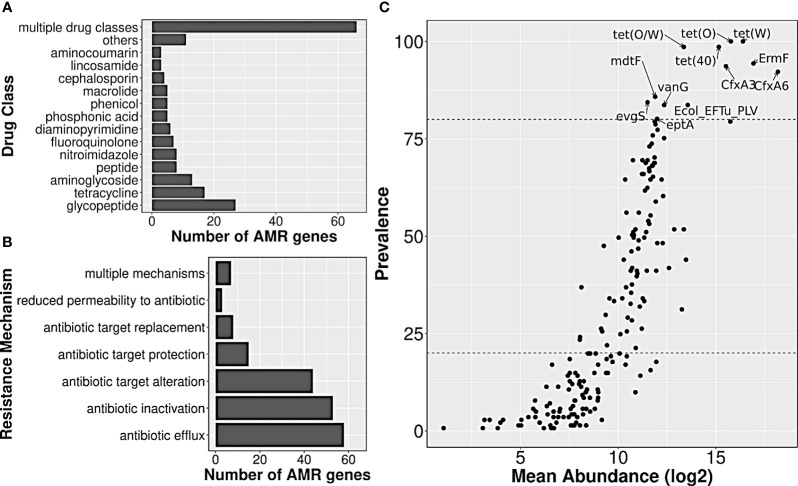
Annotation of AMR genes observed in Indian samples, and their abundance/prevalence. Bar plots showing the distribution of **(A)** drug class information of their targets, and **(B)** resistance mechanisms involved. **(C)** A scatter plot showing the abundance and prevalence of AMR genes in Indian samples. A subset of AMR genes that were highly prevalent and abundant have been labeled.

A total of 141 healthy gut metagenome samples from three cohorts, belonging to two Bioprojects with complete metadata information, were considered for the current analysis. These samples not only differed in (Indian) biogeographical locations but also differed in their lifestyle, diet, gender, and age. Data processing for read quality, adaptor content, and host contamination resulted in ~10 million reads per sample, and co-assembly of these quality-filtered WGS data yielded 6,121,077 contigs, which were further filtered based on the length, resulted in 1,540,199 contigs. AMR genes from the CARD database were searched in filtered co-assembly, and 2340 redundant AMR genes were detected, out of which 188 constituted a non-redundant AMR gene set. The majority of the genes targeted glycopeptide antibiotics (14%) followed by tetracycline (9%) and aminoglycoside (7%) antibiotics (**Figure 2A**; **Supplementary Table S2**). The identified resistome showed seven different mechanisms by which it could escape or neutralize the effect of antibiotics; few genes were involved in more than one mechanism. Among different mechanisms of action utilized by the resistome, antibiotic efflux (31%), antibiotic inactivation (28%), and target alteration (23.4%) were the three most frequent ones, whereas, reduced permeability to antibiotic was rarest (≤1%) (**Figure 2B**; **Supplementary Table S2**).

### 3.2 Antibiotics targeted by highly prevalent AMR genes showed associations with WHO AWaRe antibiotics groups

The median abundance of 188 AMR genes across all samples ranged from 0 to 319,509, with genes *nimG* and *cfxA6*, having the lowest and highest abundance, respectively. Twelve of them such as *tet(O)*, *tet(W)*, *tet(40)*, *tet(O/W)*, *ErmF*, etc., were present in ≥80% of the samples, and had high abundance values as well, with median abundance of >24,000 TPM. Such AMR genes were henceforth labeled as highly prevalent. On the other hand, almost half of the detected AMR genes (n=92) such as *nimG*, *AAC(6)-Im*, *BRP(MBL)*, *norC*, etc., had very low prevalence (<20%), and also had much lower abundance (median abundance: 0 TPM) (**Figure 2C**; **Supplementary Table S2**).

Among the antibiotics classes targeted by the highly prevalent AMR genes, tetracycline class was targeted by 4–5 genes, followed by macrolides (erythromycin, etc.) by three genes; second-generation cephalosporin (cephamycin), fluoroquinolones (norfloxacin) and penam (oxacillin, etc.) by two each; streptogramins (dalfopristin, etc.), peptides (polymyxin B), and glycopeptides (vancomycin) by one each. Modification of the target (by protection or alteration) was the most frequent mechanism of resistance, followed by antibiotic efflux (by Major Facilitator Superfamily Pump or Resistance-Nodulation-cell Division pump) and antibiotic inactivation (by Beta-lactamase). The list of targeted antibiotics was under-represented with the ‘Access’ category antibiotics (p-value: 0.06; **Supplementary Table S3**), and was close to being over-represented with the ‘Watch’ category ones (p-value: 0.1; **Supplementary Table S3**). Only two (targeted antibiotics) were in the ‘Reserve’ and/or ‘Essential’ category, namely, Polymyxin B and Dalfopristin/Quinupristin.

### 3.3 Seven ‘Reserve’ group antibiotics were targeted by a quarter of moderately or highly prevalent AMR genes

Out of a total of hundred moderately or highly prevalent AMR genes (with prevalence >=20%), and a total of twenty-nine antibiotics in the WHO AWaRe ‘Reserve’ list, twenty-seven of such genes targeted seven ‘Reserve’ group antibiotics, either exclusively or along with other antibiotic groups. The distribution of such AMR genes showed that the majority of them (almost four-fifths) had a prevalence of up to 60%, peaking in the range of 50-60% (**Supplementary Figure S1**, **Supplementary Table S4**). Those (AMR genes) exclusively targeting the ‘Reserve’ group of antibiotics include *eptB* and *ArnT* targeting colistin; *mdtG*, *Ecol_GlpT_FOF*, and *Ecol_UhpT_FOF* targeting fosfomycin; and *PmrF*, *ugd*, and *eptA* targeting Polymyxin. While *eptA* had the highest prevalence and abundance (80% and 940 TPM, respectively), pmrF had the lowest (20% and 0 TPM, respectively).

### 3.4 Location-specific prevalence trends were fewer in number

**Figure 3:**
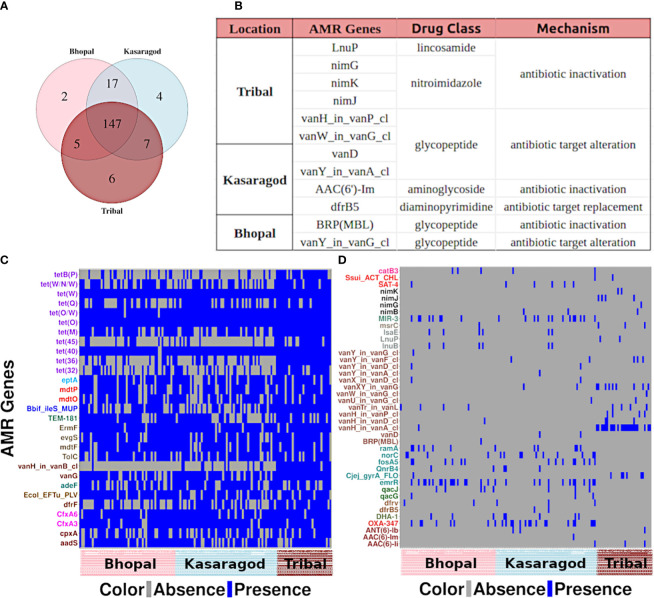
Location-specific trends of AMR genes **(A)** A venn diagram showing unique and common AMR genes across three cohorts, and **(B)** details of the genes unique to each cohort. Heatmaps showing the AMR genes that are **(C)** highly prevalent and **(D)** completely absent in at least one of the cohorts.

The AMR gene occurrences showed only a few location-specific patterns wherein the genes showed (nearly complete) presence or absence in at least one of the three cohorts. As few as two, four, and six AMR genes were uniquely present in Bhopal (central India; urban), Kasargod (south-west coastal India; urban), and tribal cohorts (north and central India), respectively (**Figures 3A, B**). To get insights into location-specific highly prevalent AMR genes, the 188 AMR genes were searched for prevalence >=80% in at least one of the cohorts, and 29 such cases were found (**Figure 3C**). For location-specific trends of very low prevalent ones, the AMR genes that were completely absent in at least one of the cohorts were also observed, and 41 such cases were there (**Figure 3D**). What emerged from the prevalence trends was that the observed AMR genes, if highly prevalent in one, also had moderate to high prevalence in other cohorts, whereas those absent in at least one cohort generally had very low prevalence in other cohorts.

From the former category, a large fraction of them (40.7%) showed resistance against the tetracycline drug class: genes *tetB(P)* and *tet(45)* from that class were highly prevalent in tribal cohorts (**Supplementary Table S2**). From the latter category, 34% of such AMR genes showed resistance against the glycopeptide class of antibiotics, in particular, against vancomycin (**Figure 3D**).

### 3.5 Tetracycline and vancomycin resistance genes were significantly abundant in tribal cohorts

**Figure 4:**
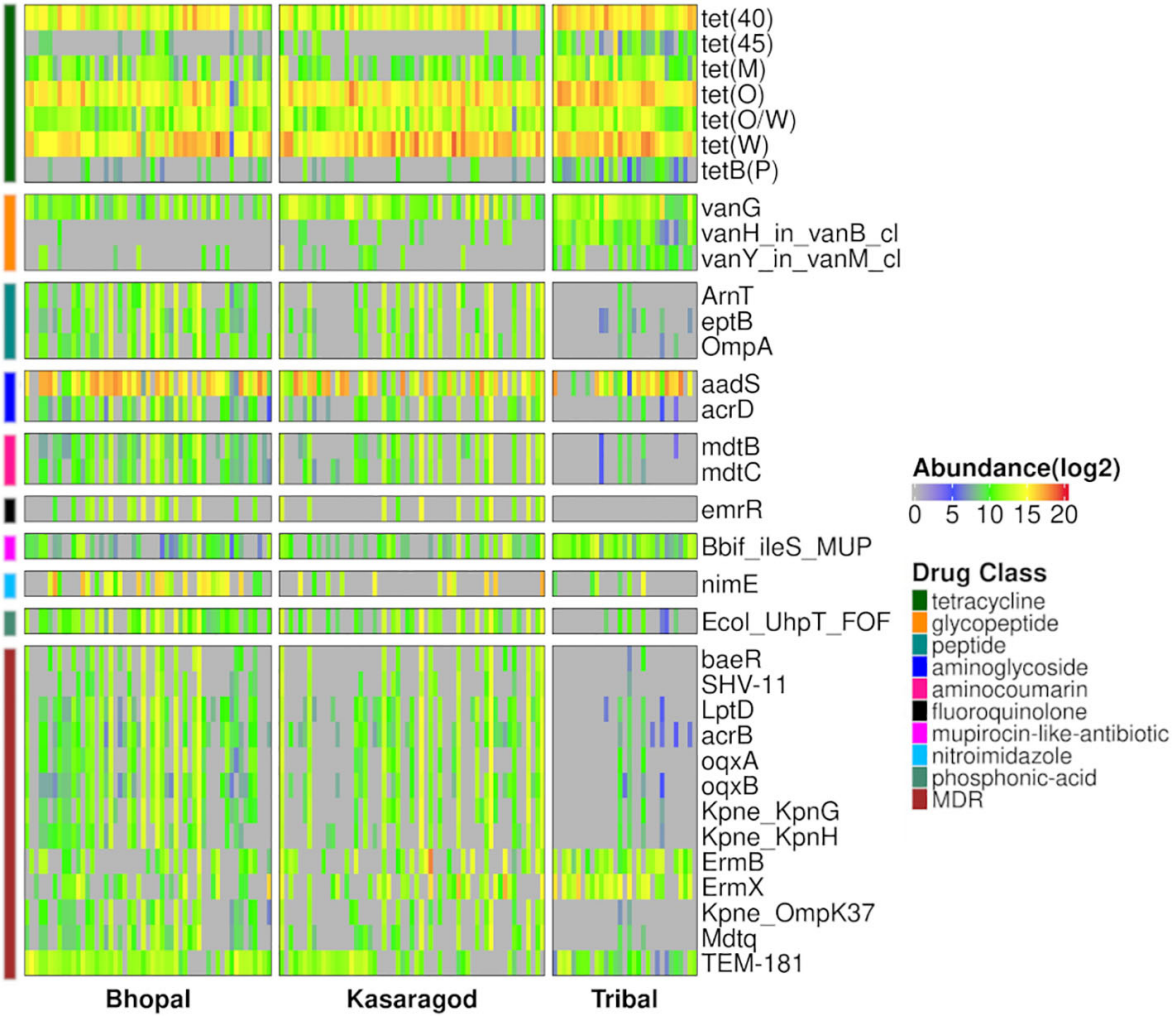
Heatmap representing prevalence and abundance of differentially abundant AMR genes in all three pairwise comparisons along with their drug class (*p-value <=0.05)*.

Differential abundance of the AMR genes was tested using two statistical models, and a total of 34 differentially abundant genes were observed in three pairwise comparisons in *both* models (p-value or p-adj<=0.05). Around half of these genes were highly abundant in the tribal cohort, and most of them belonged to the tetracycline resistance drug class (**Figure 4**; **Supplementary Table S5**). Interestingly, the dominance of tetracycline resistance in Indian tribes has been reported in an earlier study (Sethi et al., 2022). Along with tetracycline, three vancomycin resistance genes (vanG, *vanH_in_vanB_cl* and *vanY_in_vanM_cl*) were differentially abundant in Tribals (**Supplementary Tables S2**, **S5**). Since vancomycin is a “watch” category of antibiotics, resistance against it is a matter of concern. Additionally, 38% of the differentially abundant genes targeted multiple drug classes, the majority of which were significantly abundant in both urban locations- Bhopal and Kasargod (**Figure 4**; **Supplementary Table S5**).

### 3.6 Abundance of a few AMR genes was also affected by diet, age, and gender

**Figure 5:**
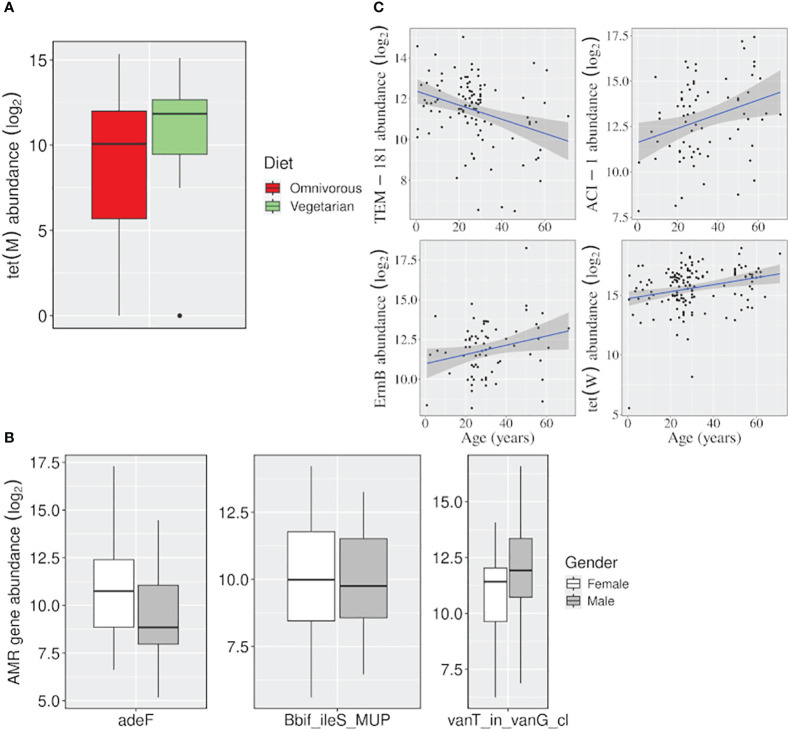
Effect of variables, other than location, on the abundance of AMR genes. Boxplots showing the AMR genes differentially abundant **(A)** between vegetarian and omnivorous dietary habits, and **(B)** between males and females. **(C)** Linear regression plots showing change in gene abundance with increasing age. (*p-value <=0.05)*.

While the geographical location has a significant impact on the abundance (or prevalence) of AMR genes, we also observed the effect of other variables such as diet, age, and gender, on a few AMR gene abundance using both negative binomial and multiple linear regression models (**Figure 5**; **Supplementary Table S6**). These variables were previously considered as covariates, but here each one was examined as the main variable, one at a time, and the effect of the remaining ones was controlled.

The *tet(M)* gene was significantly abundant among vegetarians. It uses target protection as a resistance mechanism to act against tetracycline antibiotics belonging to both ‘watch’ and ‘access’ groups. Besides, females and males showed a higher abundance of *adeF* (a membrane fusion protein) and *vanT_in_vanG_cl* genes, which target the tetracycline/fluoroquinolone and glycopeptide class of antibiotics, respectively. Even for the ‘age variable, a significant decrease in the abundance of a beta-lactamase gene *TEM-181*, which could target ceftazidime (cephalosporin class; ‘reserve’ antibiotic), with age was observed (**Figure 5**; **Supplementary Table S2**).

### 3.7 A substantially higher proportion of AMR genes were present in binned set (MAGs) than in unbinned

**Figure 6:**
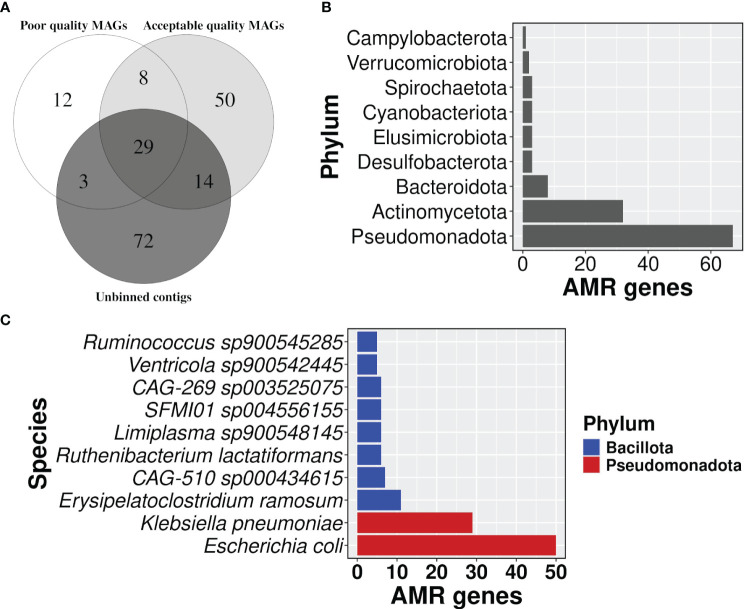
Distribution of AMR gene carrying contigs. **(A)** A venn diagram showing the proportion of AMR gene carrying contigs in each group i.e, acceptable quality MAGs, poor quality MAGs, and unbinned contigs, **(B)** a bar plot representing the number of AMR genes in each phylum, and **(C)** top 10 bacterial species in carrying the number of AMR genes.

Binning of filtered co-assembly resulted in 631 MAGs, which included 122,807 contigs. However, a relatively larger fraction of the contigs of the co-assembly remained unbinned (1,417,392). MAGs were further filtered for completeness >= 50% and contamination <= 10%, and 367 acceptable-quality MAGs were obtained. The AMR-carrying contigs in the co-assembly were traced to one of the three groups namely acceptable-quality MAGs, poor-quality MAGs, and unbinned contigs, which had about 1005, 325, and 988 AMR-carrying contigs respectively. Despite the disproportionately smaller size of the binned set, they contained a substantially higher proportion of AMR-carrying contigs than the unbinned set (*p-value*: 2.2E-16) Even after correcting for redundancy among the AMR genes in each set, the difference in the proportion of non-redundant AMR genes in two sets still followed the same trend as observed above (**Figure 6A**; **Supplementary Table S7**).

### 3.8 Taxonomy assignment showed that *Escherichia coli* carried the highest AMR gene burden

Taxonomic profiles showed that phylum Pseudomonadota had the highest number of AMR genes followed by Actinomycetota (**Figure 6B**; **Supplementary Table S8**). The detection of Cyanobacteriota in the human gut was surprising. But it’s known that Vampirovibrionia (formerly Melainabacteria) is one of the closest non-photosynthetic living relatives of Cyanobacteriota, which also includes gut symbionts (Grettenberger et al., 2020; Oliver et al., 2021). In our analysis, Vampirovibrionia was classified as a class under phylum Cyanobacteriota. Species *Escherichia coli* carried the highest number of AMR genes (n=50) in its genome, followed by *Klebsiella pneumoniae* with 29 genes (**Figure 6C**; **Supplementary Table S8**), and both of the species belong to the phylum Pseudomonadota. In line with the AMR surveillance report 2021, MAGs belonging to both *E. coli* and *K. pneumoniae* showed resistance to broad-spectrum antibiotics fluoroquinolone, third-generation cephalosporine, carbapenem, and colistin, which is a last resort antibiotic **Supplementary Table S8**) (AMR Surveillance Network, 2021).

### 3.9 A fraction of AMR genes were part of mobile genetic elements

**Figure 7:**
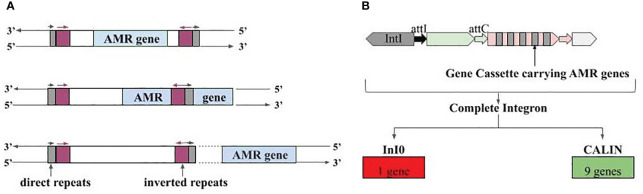
Association of AMR genes with mobile genetic elements. **(A)** Three different types of associations with IS elements, i.e., IS elements carrying the AMR genes fully, IS element overlapping with AMR genes, and AMR genes present at the vicinity of IS element, **(B)** Integron gene cassettes identified in AMR gene carrying contigs; InI0 is Integron-integrase lacking cassette and CALIN is Cluster of attC sites lacking integron integrase.

The AMR gene-carrying contigs were also searched for mobile genetic elements i.e., IS elements, integrons, and ICEs. The positive results were further checked for their complete or partial overlap with AMR genes based on coordinates. For IS elements, three different cases were observed where 1) the AMR gene was present within the IS element, 2) the AMR gene and IS element were partially overlapping, and 3) the AMR gene in the flanking region or far apart from the IS element (**Figure 7A**). Case 1 included gene *vanY_in_vanB_cl*, which targets vancomycin (‘watch’ group), gene *QnrS1*, which targets antibiotics of fluoroquinolone class (‘watch’ group), and gene *lnuC*, which targets lincosamide antibiotic (‘watch’ group). All of them were present in the contigs of plasmid origin and could transmit easily. Gene *adeF*, *vanW_in_vanI_cl*, *tet(45)*, and 14 more genes were part of case 2. For case 3, we had 45 such contigs where the IS element and AMR genes were separate entities (**Supplementary Table S9**).

Besides IS elements, we also observed the presence of AMR genes in partial Integrons i.e., ‘Integron-integrase lacking cassette’ (InI0) and ‘Cluster of attC sites lacking integron integrase’ (CALIN). CALIN had nine AMR genes showing complete overlap, and these genes can target antibiotics belonging to the ‘access’ and ‘watch’ group. Gene *OXA-1* targets carbapenem class of antibiotics (‘access’ and ‘watch’ group), *aadA5* targets aminoglycoside antibiotic (‘access’ and ‘watch’ group), and *AAC(6’)-Ib-cr6* targets fluoroquinolone, and aminoglycoside antibiotic (‘access’ and ‘watch’ group) (**Figure 7B**; **Supplementary Table S10**). Lastly, one more type of mobile genetic element namely Integrative and conjugative elements, was also found in close vicinity of a few AMR genes. Tetracycline targeting genes *tet(32)* (‘access’ and ‘watch’ group) and *tet(45)* (‘access’ group), vancomycin (‘watch’ group) targeting *vanW_in_vanI_cl* gene antibiotic etc., were among them (**Supplementary Table S11**).

The occurrence of the HGT event was also indicated for two vancomycin-resistant genes namely *vanY_in_vanB_cl* and vanY_in_vanG_cl. Identical sequence of gene *vanY_in_vanB_cl* between two species of Bcillota phylum, between two pairs of unbinned contigs were observed. Similarly, the identical sequence of vanY_in_vanG_cl was observed between poor-quality bin and unbinned contig.

## 4 Discussion

### 4.1 First report on AMR genes in healthy gut microbiome samples from multiple locations of India

In the present study we have analyzed the publicly available fecal WGS sequencing data to assess the AMR load in gut microbiomes of healthy Indian cohorts. This is the first report on healthy Indian gut resistome where a total of 141 samples were taken from three different Indian locations and belonged to urban and tribal groups. We observed the presence of a total of 188 AMR genes which can target several drug classes (**Figure 2A**), either uniquely or along with other genes in all three cohorts. For instance, the moderately to highly prevalent ones can target seven ‘reserve’ group antibiotics which also included the essential ones. The geographic location/lifestyle majorly accounted for resistome composition variability, which was also reported in a study involving global samples (Qiu et al., 2020), along with other factors such as age, gender, and diet. Interestingly, age significantly affected the abundance of four AMR genes (*TEM-181, EmrB, ACI-1*, and *tet(W)*), and we reason that this could be due to the differential use of antibiotics in different age groups (Patangia et al., 2024).

### 4.2 A higher abundance of tetracycline resistance genes synced with the global data, but that of vancomycin resistance genes in Indian tribal cohorts was intriguing

We observed the resistance majorly against the glycopeptide antibiotic drug class followed by tetracycline and aminoglycosides in all cohorts (**Figure 2A**). These results were in sync with the previous studies on healthy Indian gut resistome which showed the dominance of tetracycline resistant genes (Bag et al., 2019; Monaghan et al., 2020; Sethi et al., 2022), and the same was true for several studies involving global samples (Ghosh et al., 2013; Hu et al., 2013; Qiu et al., 2020). The higher abundance of glycopeptides (Vancomycin) in overall Indian cohorts and their differential abundance in tribal cohorts was intriguing to us, as they have such a higher prevalence. A previous study also reported the presence of vancomycin resistance genes (*vanA*, *vanB*, and *vanC*) in healthy Indian tribal cohorts (Sethi et al., 2022). It is known that bacteria such as *Lactobacillus* have intrinsic resistance to Vancomycin (Gueimonde et al., 2013), and the tribal cohort might harbor several such bacterial species.

Similar to resistance profiles of healthy individuals, isolated genomes of pathogenic bacteria from acute diarrheal patients from India showed resistance genes against β-lactam, aminoglycoside antibiotics, and also possessed multiple multidrug resistance efflux pumps (Kumar et al., 2017).

### 4.3 The highest AMR gene burden of *E. coli* in both Indian and global cohorts may pose a risk of becoming a transmission hub

Among all the MAGs identified in our data, *E.coli* had the highest load of AMR genes (n=50). Along with ‘access’ group antibiotics, it can also impart resistance against the ‘reserve’ group (polymyxin, ceftazidime, imipenem, fosfomycin, tigecycline) and ‘watch’ group antibiotics (vancomycin, norfloxacin, ciprofloxacin etc) (**Supplementary Table S2**; **Supplementary Table S8**). *E. coli* isolates detected from 2009-2015 from diarrheal patients showed higher resistance genes against the ‘access’ group (gentamycin, ampicillin, trimethoprim, nalidixic acid), ‘watch’ group (kanamycin, streptomycin, ciprofloxacin), and low detection rate was observed against spectinomycin and polymyxin B (Kumar et al., 2017). From 2016 to 2021, a decreasing trend of susceptibility of *E. coli* was observed for imipenem (85.9% to 64%), meropenem (80.7% to 695%), ceftazidime (25% to 18%), ciprofloxacin (20.3% to 19%), and amikacin (83.8% to 78.2%) (Inda-Díaz et al., 2023). Unfortunately, the resistance against colistin, which is a last resort antibiotic of human medicine was observed in our data and ICMR surveillance report (AMR Surveillance Network, 2021). An AMR gene *Ecol_GlpT_FOF* in *E. coli*, which potentially targets fosfomycin showed partial overlap with IS element. This makes it more likely to get transmitted to other members of the gut community. Fortunately, we didn’t find an association of the remaining AMR gene from *E. coli* MAG with any mobile genetic element.

Having a higher abundance of resistance genes is also associated with a higher abundance of the species harboring them. The previous analysis of the same data showed a mean relative abundance of *E. coli* >= 0.1% (Chandel et al., 2023). The co-occurrence network between AMR genes and microbial taxa in global cohorts clearly showed *E. coli* as a hub (Qiu et al., 2020). Taken together, *E. coli* being an AMR gene hub, with higher abundance in the gut poses a threat to AMR transmission in the gut community.

### 4.4 Association of AMR genes with mobile elements poses a risk for transmission to other gut bacteria

The CARD database for AMR genes (Alcock et al., 2020) annotated at least two tetracycline resistance genes, *tet(O)* and *tet(W)*, having association with conjugative plasmids or conjugative DNA (**Supplementary Table S2**). Moreover, the results on ICEs in Indian gut metagenomes showed evidence of association of another two genes, *tet(45)* and *tet(32)*, with the conjugative elements (**Supplementary Table S11**). These two genes were moderately to highly prevalent (99% and 48%, respectively). These pieces of evidence suggest that conjugative DNA or plasmids might have been a key factor behind the high prevalence of these AMR genes, in particular, the tetracycline resistance genes. However, there were exceptions too, as one of the vancomycin resistance genes (*vanW_in_vanI_cl*), which also was detected as part of ICEs, had a very low prevalence (5%).

Results of association with another mobile element, namely IS elements, showed that multiple vancomycin resistance genes (*vanY_in_vanB_cl*, *vanW_in_vanI_cl*, *vanG*, *vanT_in_vanG_cl, and vanY_in_vanA_cl*) overlapped, completely or partially, with the composite transposons (**Supplementary Table S9**), and among them, *vanG* and *vanT_in_vanG_cl* showed higher abundance (84% and 41%, respectively) as compared to others which were <15%. This suggests that a subset of vancomycin resistance genes used IS elements/DNA transposons for faster spread across bacteria and/or individuals. Even few other AMR genes (such as OXA-1 beta-lactamase, *catB3* Chloramphenicol acetyltransferase) likely used integrons for their transmission, a majority of them however had lower prevalence in Indian samples (**Supplementary Table S10**), which was in contrast to a recent finding where integrons were demonstrated to accelerate the evolution of AMR (Souque et al., 2021). This difference however can be due to the difference in the level of selection pressure applied. As HGT occurs more frequently between ecologically similar bacteria than phylogenetically related ones (Jansen and Aktipis, 2014), thus a narrow niche formed in the gut acts as a reservoir of AMR genes for commensals as well as pathogens.

The association of vancomycin-resistant gene *vanY_in_vanB_cl* with IS element and its recent exchange between two species of Bacillota phylum further confirms its chances of getting spread to the gut community.

### 4.5 Limitations and future directions

One of the major limitations of the study is the representation of samples. Only three cohorts covering 141 samples qualified the criteria set for inclusion in the study, which is not a sound representation of the Indian population. The availability of more gut metagenome samples of Indians in the future may bring out trends with better robustness. The other limitation of the study is that the large chunk of assembly remained unbinned. While we searched for AMR genes in each assembled contig, the bacterial source of unbinned AMR-carrying contigs remained unknown.

Besides, *E. coli* has a relatively modest abundance in healthy Indian gut (Chandel et al., 2023). Since it was observed to carry the highest AMR genes in its genome, it will be interesting to experimentally investigate the extent of horizontal transfer of AMR genes from *E. coli* to other gut bacteria. Likewise, we also advocate for further experimental studies on reasons for the high prevalence of vancomycin resistance genes in Indian tribal cohorts.

## Data availability statement

The original contributions presented in the study are publicly available. This data can be found here: NCBI, accession PRJNA397112, PRJNA531203.

## Ethics statement

Ethical approval was not required for the study involving humans in accordance with the local legislation and institutional requirements. Written informed consent to participate in this study was not required from the participants or the participants’ legal guardians/next of kin in accordance with the national legislation and the institutional requirements.

## Author contributions

NC: Formal analysis, Investigation, Methodology, Software, Writing – original draft, Writing – review & editing, Conceptualization, Data curation, Visualization. JG: Software, Conceptualization, Data curation, Methodology, Writing – review & editing. VT: Conceptualization, Investigation, Project administration, Supervision, Writing – original draft, Writing – review & editing, Methodology, Resources.

## Supplementary material

Supplementary Figure 1 Distribution of prevalence of AMR genes targeting the ‘Reserve’ group of antibiotics.
