# Supplementary figures and images for: Antimicrobial resistance burden, and mechanisms of its emergence in gut microbiomes of Indian population
Source: Front Microbiomes. 2024 Jul 18;3:1432646. doi: 10.3389/frmbi.2024.1432646 (PMC12993557; doi:10.3389/frmbi.2024.1432646)

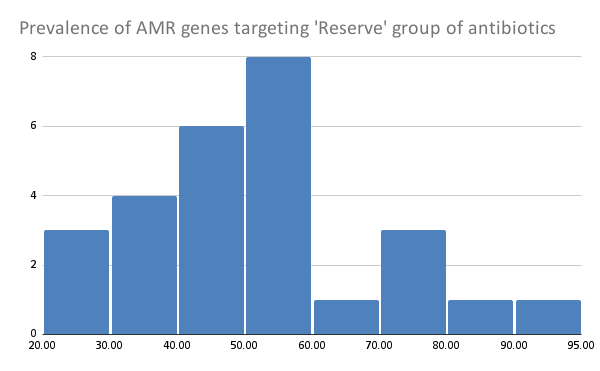

Supplement: Supplementary Figure 1 — Distribution of prevalence of AMR genes targeting the ‘Reserve’ group of antibiotics. [file Image_1.tif]
